# Disc Battery - An Unusual Vaginal Foreign Body in a Child

**DOI:** 10.21699/ajcr.v7i4.467

**Published:** 2016-09-01

**Authors:** Yousuf Aziz Khan, Mansoor Mahmood, Esmaeel Taqi

**Affiliations:** Department of Paediatric Surgery, Ibn Sina Hospital of Surgical Specialties, Al-Sabah Health Region,Safat – 13115, State of Kuwait.

**Keywords:** Vaginal foreign body, Children, Lithium disc battery

## Abstract

Disc battery ingestion and esophageal injury is well-known in children. Insertion of a disc/lithium battery into body’s natural orifices is rarely reported. We present a case of self-insertion of a lithium battery into the vagina by a 2 ½ year old female. Vaginoscopy was performed and the battery was retrieved which had corroded and caused vaginal ulceration. Post-operative outcome was favorable. Treating physicians must be aware of the hazardous effects of insertion of lithium batteries as it may cause significant damage in a short period.

## INTRODUCTION

Foreign body (FB) ingestion or insertion into the body’s natural orifices is one of the common presentations encountered in paediatric surgical practice. In prepubertal girls with vaginal discharge, an incidence of approximately 4-10% vaginal FB has been reported.[1] The commonly inserted vaginal FB by children include small nuts, safety pins, beads, plastic stoppers, crayons, pencils, material from cloths and carpet. However, pieces of toilet paper are the commonest.[1,2] Vaginal self-insertion of a lithium battery is rarely seen. Herein we report a female child who put in a disc battery into her vagina which was successfully retrieved.

## CASE REPORT

A 2½ year old female child was referred to us from a primary care center with a history of self-insertion of a lithium disc battery into the vaginal orifice. The event occurred eight hours prior to presentation and was noticed by the care-giver. She had no urinary symptoms. On examination, she had stable vitals and, general and systemic examination was unremarkable. On perineal examination, blackish discharge was noticed from the vaginal orifice with some erythema around it. Plain abdomino-pelvic x-ray revealed a round radio-opaque object in the pelvic region, compatible with a button battery.(Fig.1a and 1b)

**Figure:**
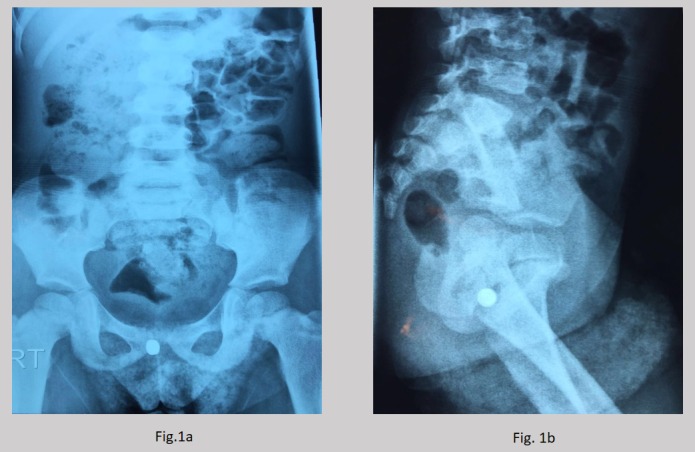
Figure 1(a, b): Plain X-ray Abdomen and Pelvis AP and lateral views: showing opacity in the perineal region.

After preparation, examination under general anesthesia and vaginoscopy was performed. Thick black paste like material was noticed inside vagina. At vaginoscopy, a lithium disc battery was found which was retrieved easily. After irrigation with normal saline, a superficial ulcer on anterior and a deep circular ulcer on the posterior vagina wall were seen about ½ cm from the hymen.(Fig.2) Irrigation with povidone-iodine solution was done and gentamicin ointment was instilled inside vagina. Subsequent proctoscopy showed an intact rectum. Postoperative course was uneventful and she was discharged home on fourth postoperative day. After 4 weeks, an examination under anesthesia was repeated with vaginoscopy and proctoscopy, which showed well healed vaginal ulcer and no adhesions and, intact rectum. She is still being followed in the out-patient clinic.

**Figure:**
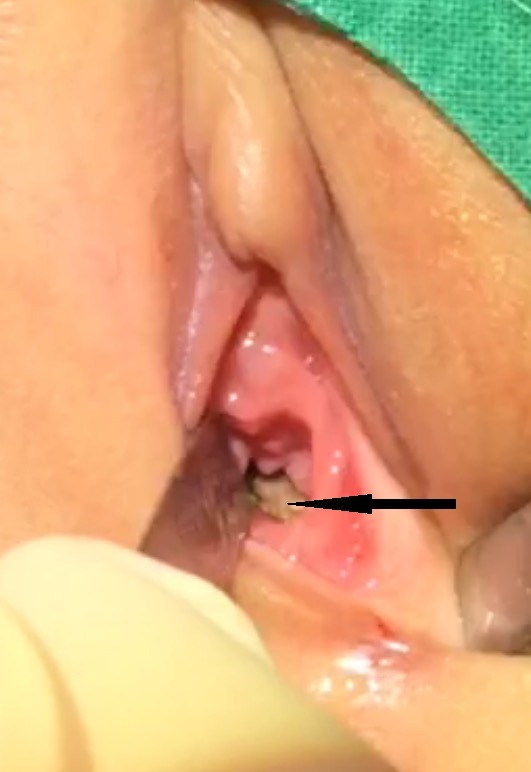
Figure 2:Deep ulcer (pointer) – at the posterior vaginal wall as visualized during examination.

## DISCUSSION

Vaginal FB is uncommon and a variety of FBs have been documented. Usually they are seen between the ages of 3 and 9 years and in 80% of the cases it is the toilet tissue paper. In our case it was a lithium disc battery which was self-introduced by the baby. Closson FT et al suggested that child / sexual abuse should not be forgotten in case of a vaginal FB in prepubertal girl.[3]

A variety of symptoms may be seen in case the event is unnoticed. Vulvo-vaginitis, vaginal bleeding, blood tinged / malodorous vaginal discharge, dysuria with lower abdominal pain, all may occur with a disc battery as with other long standing vaginal FBs.[4,5] Our patient had a definitive history of battery insertion and clinically she had blackish discharge per vagina and erythema around.

Vaginal disc battery insertion may produce severe tissue damage if not retrieved well in time. Superficial to deep vaginal mucosal ulceration, full thickness necrosis and urinary or fecal fistula may occur depending upon the duration and pH of the alkaline battery. The physiological low pH of vagina may play a defensive part reducing the severity of burns.[1] Peroperatively our patient had ulcerations at the vaginal walls and an intact rectum. Timely presentation and the protective pH of vagina had possibly saved the child from severe injury. Long-term follow-up is suggested to ensure complete vaginal healing without any fistula and to record complications of vaginal stenosis, intra-uterine synechiae, and pelvic adhesions.[4] Children should not be given toys with disc batteries and strict observation should be made if such toys are used.

## Footnotes

**Source of Support:** Nil

**Conflict of Interest:** None declared

